# Antimicrobial susceptibility and resistance mechanisms of methicillin resistant *Staphylococcus aureus* isolated from 12 Hospitals in Turkey

**DOI:** 10.1186/s12941-014-0044-2

**Published:** 2014-09-16

**Authors:** Ömer Yıldız, Ahmet Yılmaz Çoban, Aslı Gamze Şener, Seher Ayten Coşkuner, Gülçin Bayramoğlu, Hüseyin Güdücüoğlu, Mustafa Özyurt, Müşerref Tatman-Otkun, Nihal Karabiber, Nuri Özkütük, Orhan Aktepe, Serkan Öncü, Uğur Arslan, Bülent Bozdoğan

**Affiliations:** ADU BILTEM Epidemiology Unit, Aydin, Turkey; Medical Faculty Medical Microbiology Department, Ondokuz Mayıs University, Samsun, Turkey; Katip Çelebi University, Atatürk Training and Research Hospital, Izmir, Turkey; Bozyaka Training and Research Hospital, Izmir, Turkey; Medical Faculty Medical Microbiology Department, Karadeniz Technical University, Trabzon, Turkey; Medical Faculty Medical Microbiology Department, Yüzüncü Yıl University, Van, Turkey; GATA Haydarpaşa Training Hospital, Istanbul, Turkey; Medical Faculty Medical Microbiology Department, Canakkale Onsekiz Mart University, Çanakkale, Turkey; Yüksek Ihtisas Hospital, Ankara, Turkey; Medical Faculty Medical Microbiology Department, Celal Bayar University, Manisa, Turkey; Medical Faculty Medical Microbiology Department, Afyon Kocatepe University, Afyon, Turkey; Medical Faculty Infectious Disease and Clinical Microbiology Department, Adnan Menderes University, Aydın, Turkey; Medical Faculty Medical Microbiology Department, Selçuk University, Konya, Turkey; Medical Faculty, Medical Microbiology Department, Adnan Menderes University, 09010 Aydin, Turkey

**Keywords:** Staphylococci, MRSA, Antimicrobial susceptibility, Resistance mechanisms, PCR

## Abstract

**Introduction:**

Methicillin-resistant *Staphylococcus aureus* (MRSA) is one of the most important nosocomial pathogens and is also emerging in Turkish hospitals. The aim of this study was to determine the antimicrobial susceptibility profiles of MRSA isolated from Turkish hospitals.

**Materials and methods:**

A total of 397 MRSA strains isolated from 12 hospitals in Turkey were included to present study. Antimicrobial susceptibilities were tested using agar dilution method. Presence of *ermA, ermB, ermC, msrA, tetM, tetK, linA* and *aac-aph* genes were studied by PCR.

**Results:**

All strains were susceptible to vancomycin and linezolid. The susceptibility rates for fusidic acid, lincomycin, erythromycin, tetracyclin, gentamycin, kanamycin, and, ciprofloxacin were 91.9%, 41.1%, 27.2%, 11.8%, 8.5%, 8.3% and 6.8%, respectively. Lincomycin inactivation was positive for 3 isolates. Of 225 erythromycin resistant isolates 48 had *ermA*, 20 had *ermC*, and 128 had *ermA-C*. PCR was negative for 15 strains. Of 3 isolates with lincomycin inactivation one had *linA* and *msrA*. Of 358 gentamycin resistant isolates 334 had *aac-aph* and 24 were negatives. Among 350 tetracyclin resistant isolates 314 had *tetM*. Of 36 *tetM* negative isolates 10 had *tetK*.

**Conclusion:**

MRSA isolates from Turkish hospitals were multiresistant to antimicrobials. Quinolone and gentamycin resistance levels were high and macrolide and lincosamide resistance were relatively low. Susceptibility rates for fusidic asid were high. Linezolide and vancomycin resistance are not emerged. The most common resistance genes were *ermA*, *tetM* and *aac-aph*. Evolution of antimicrobial susceptibilities and resistance genes profiles of MRSA isolates should be surveyed at regional and national level for accurate treatment of patients and to control dissemination of resistance genes.

## Introduction

Staphylococci are important infection agents that cause hospital and community acquired infections. These bacteria have ability to adapt themselves to difficult conditions and successful clones have capacity of epidemic and pandemic dissemination [1]. Increasing resistance problem in staphylococci became an important public health problem. In 1944 when penicillin became available for use the susceptibility rate of *Staphylococcus aureus* to penicillin was >94% which became <5% recently [2]. Methicillin resistance appeared and started to disseminate from 1980 and became one of the major problem in hospital infections. Methicillin resistance is due to acquisition of a transpeptidase, PBP2a, involved in cell wall synthesis that has low affinity for beta lactam antibiotics which rends bacteria resistant to all beta lactam antibiotics. Treatment of infections due to methicillin resistant *S. aureus* (MRSA) causes problems due to restricted number of choices [1]. Especially from 2003, when vancomycin resistant *S. aureus* emerged it became urgent to search new treatment possibilities for these bacteria [3]. In addition emergence and dissemination of community MRSA isolates forced to evaluate empiric treatment options in consideration with changing resistance profiles of these bacteria. MRSA strains do not affect only human but also infect farm animals and pets [4]. Although development of new antibiotics reduced dramatically recently, some antibiotics like daptomycin, linezolid and tigecyclin could be commercialized lately [1].

In the present study susceptibilities of 397 MRSA isolated from 12 centers in Turkey to linezolid, fusidic acid, kanamycin, gentamycin, erythromycin, lincomycin, tetracyclin, vancomycin and ciprofloxacin were tested by agar dilution method and presence known resistance genes were verified by PCR using specific primers.

## Materials and methods

A total of 12 centers from 11 cities participated to the present study and sent MRSA isolates to Aydın where susceptibility testing and molecular studies were done at ADU BILTEM Epidemiology Unit. Methicillin resistance was confirmed by cefoxitin disc method. A total of 397 MRSA isolates were collected from hospitalized patients between 2006–2008, from Aydın (15 isolates), Izmir (2 centres 17 and 22 isolates), Afyon (32), Manisa (23), Van (42), Trabzon (54), Samsun (51), Ankara (31), Konya (28), Istanbul (55), and Edirne (36).

### Determination of antimicrobial susceptibilities

#### Agar dilution method

Antibiotics tested were linezolid, fusidic acid, kanamycin, gentamycin, erythromycin, lincomycin, tetracyclin, vancomycin and ciprofloxacin. Erythromycin and fusidic acid were from Koçak Farma (Tekirdağ, Türkiye), kanamycin, tetracyclin and vancomycin were purchased from Sigma, and commercial injectable preparations were used for the remaining antimicrobials. Agar dilution method was used as described previously [5]. Shortly plates were prepared with serial dilution from 64 or 128 mg/L antibiotic concentrations. Inoculum with 5X10^4^ bacteria was placed onto agar using multipoint inoculator. After 16–20 h incubation at 37°C the lowest concentration that inhibits bacterial growth was accepted as MIC. Reference strain *S. aureus* RN4220 was included to each run.

#### Gots test

All lincomycin resistant isolates were tested by Gots’ test for presence of resistance by antibiotic inactivation. For this purpose to 19 ml agar at 50-60°C 19 ml BHI (Brain Heart infusion) agar 0,5 mg/L clindamycin and 1 ml overnight broth of *Micrococcus luteus* ATCC9341 were added, mixed and poured to petri dish and left for solidification. The MRSA isolates were inoculated as small round onto agar. On one plate approximately 20 MRSA isolates were inoculated. After 24 h incubation at 37°C plates were left 24 h at room temperature. Growth of indicator bacteria in the round of tested bacteria was accepted as positive which showed presence of resistance mechanism by inactivation [6].

### Determination of resistance mechanisms

#### DNA extraction

DNA extraction was done using Instagen Matrix (BioRad) as recommended by manufacturer. Shortly 1–2 colonies were homogenized in 1 ml of distillated water and centrifuged at 10000 rpm for 1 minute. Supernatant were discarded and pellet was homogenized with 100 μl of instagen matrix. After incubation at 55°C during 15–30 min the mixture was vortexed and incubated at 95°C during 8 min. Lysate were centrifuged and 2 μl of supernatant were used as DNA for PCR reactions.

#### PCR

Erythromycin, lincomycin, gentamycin and tetracyclin resistant MRSA isolates were tested for the presence of *msrA*, *ermA*, *ermB*, *ermC*, *linA, linB*, *aac-aph, tetM* and *tetK* genes by PCR using specific primers. List of the primers and PCR conditions are shown in Table 1 [7-11].

**Table 1:** **Primers and PCR conditions used to amplify resistance genes**

**Resistance genes**	**Primers**	**PCR conditions**	**References**
*ermA*	F 5′TCT AAA AAG CAT GTA AAA GAA3′	Pre cycle 93°C 3 min,	[7] Sutcliffe 1996
	R 5′CTT CGA TAG TTT ATT AAT ATT AGT3′	35 cycles: 93°C 60 s, 52°C 60 s, 72°C 60 s	
*ermB*	F 5′GAA AAG GTA CTC AAC CAA ATA3′	Last cycle 72°C 5 min	
	R 5′AGT AAC GGT ACT TAA ATT GTT TAC3′		
*ermC*	F 5′GCT AAT ATT GTT TAA ATC GTC AAT TCC3′		
	R 5′GGA TCA GGA AAA GGA CAT TTT AC3′		
*msrA*	F 5′GCA AAT GGT GTA GGT AAG ACA ACT3′	Pre cycle 93°C 3 min,	[7] Sutcliffe 1996
	R 5′ATC ATG TGA TGT AAA CAA AAT3′	35 cycles: 93°C 30 s, 52°C 30 s, 72°C 60 s	
		Last cycles 72°C 10 min	
*linA*	F 5′GTA TTA ACT GGA AAA CAG CAA AG3′	Pre cycle 5 dk 94°C	[10] Lina 1999
	R 5′GAG CTT CTT TTG AAA TAC ATG G3′	35 cycles 45 s 94°C, 45 s 54°C,1 min at 72°C	
*linB*	F 5′CCTACCTATTGTTTGTGGAA 3′	Last cycle 5 min at 72°C	[11] Bozdogan 1999
	R 5′ATAACGTTACTCTCCTATTC 3′		
*tetM*	F 5′GTG GAC AAA GGT ACA ACG AG3′	Pre cycle 93oC’de 5 dk	[9] Warsa 1996
	R 5′CGG TAA AGT TCG TCA CAC AC3′	35 cycles 93°C 60 s, 52°C 60 s, 72°C 60 s	
*tetK*	F 5′CAG CAG ATC CTA CTC CTT3′	Last cycle 10 min at 72°C	
	R 5′TCG ATA GGA ACA GCA GTA3′		
*aac-aph*	F 5′GAG CAA TAA GGG CAT ACC AAA AAT C3′	Pre cycle 94C’de 5 dk,	[8] Kao 2000
	R 5′CCG TGC ATT TGT CTT AAA AAA CTG G3′	35 cycles 94°C 30 s, 50°C 30 s, 72°C 30 s	
		Last cycle 7 min at 72°C	

## Results

### Susceptibilities to antibiotics

MICs and resistance was evaluated using CLSI criteria [12]. All 397 MRSA isolates tested were found to be susceptible to vancomycin and linezolid. Only 8 of 397 MRSA isolates were susceptible to all antibiotics tested. In Table 2 MIC_50_ and MIC_90_ of the isolates are shown for each antibiotic tested. The number of resistant isolates to erythromycin, lincomycin, tetracyclin, gentamycin, fusidik acid, ciprofloxacin and kanamycin were 225 (%56.7), 168 (%42.3), 350 (%88.2), 358 (%90.2), 32 (%8.1), 366 (%92.2), and 363 (%91.4), respectively (Table 3). Distribution of resistance levels for the antibiotics for each centre is shown at Table 4.

**Table 2:** **MIC**_**50**_ **, and MIC**_**90**_ **values for antibiotics tested for MRSA isolates**

**Antibiotics**	**MIC**_**50**_ **(mg/L)**	**MIC**_**90**_ **(mg/L)**
Tetracyclin	128	128
Ciprofloxacin	>64	>64
Linezolid	2	2
Fusidic Acid	0.25	0.5
Vancomycin	1	2
Kanamycin	>128	>128
Erythromycin	16	>128
Lincomycin	1	>128
Gentamycin	64	128

**Table 3:** **Prevalence of resistance rates of 397 MRSA isolates**

**Antibiotics**	**Number of isolate (%)**		
	**Resistance rates**	**Intermediate resistance rates**	**Susceptibility rates**
Erythromycin	225 (56.7)	64 (16.1)	108 (27.2)
Lincomycin	168 (42.3)	66 (16.6)	163 (41.1)
Tetracyclin	350 (88.2)	0	47 (11.8)
Ciprofloxacin	366 (92.2)	4 (1)	27 (6,8)
Kanamycin	363(91.4)	1 (0.3)	33 (8.3)
Gentamycin	358 (90.2)	5 (1.3)	34 (8.5)
Fusidic Acid	32 (8.1)	0	365 (91.9)
Linezolid	0	0	397 (100)
Vancomycin	0	0	397 (100)

**Table 4:** **Resistance rates by centre of MRSA isolates**

	**% of resistant isolates**								
**Centre (No of isolates)**	Tetra**	**Cipro**	**Line**	**F. Acid**	**Vanco**	**Kana**	**Erythro**	**Linco**	**Genta**
Aydın (15)	86.6	100	0	0	0	100	80	60	93.3
İzmir (17)A*	94.1	100	0	5.8	0	100	52.9	64.7	88.2
İzmir (22)B*	95.4	100	0	9.1	0	90.9	72.7	63.6	90.9
Afyon (23)	91.3	86.9	0	21.7	0	86.9	21.7	26.1	82.6
Manisa (23)	95.6	86.9	0	0	0	95.6	69.5	56.5	91.3
Van (42)	95.2	97.6	0	2.4	0	95.2	4.7	2.4	92.8
Trabzon (54)	88.9	74.1	0	12.9	0	70.3	52.7	40.7	68.5
Samsun (51)	98	94.1	0	7.8	0	92.1	45.1	31.3	90.2
Ankara (31)	100	100	0	0	0	96.7	58	41.9	80.6
Konya (28)	92.8	96.4	0	10.7	0	96.4	89.3	46.2	92.8
İstanbul (55)	52.7	96.3	0	3.6	0	96.3	56.3	58.2	96.3
Edirne (36)	88.8	88.8	0	8.3	0	88.8	50	41.6	88.8
Toplam (397)	88.2	92.2	0	8.1	0	91.4	56.7	42.3	90.2

*A: Katip Celebi University Bozyaka Hospital, B: Izmir Atatürk State Hospital.

**Tetra, Tetracycline; Cipro, Ciprofloxacin; Line, Linezolid; F. Acid, Fusidic Acid; Vanco, Vancomycin; Kana, Kanamycin, Erythro, Erythromycin; Linco, Lincomycin; Genta; Gentamycin.

### Resistance mechanisms

Of 225 erythromycin resistant MRSA isolates 48 carried *ermA*, 20 carried *ermC*, 1 carried both *ermA* and *ermB*, 1 carried both *ermB* and *ermC*, 128 carried both *ermA* and *ermC*, 2 carried *ermA*, *ermB* and *ermC*, 2 carried *msrA*, 2 carried *msrA* and *ermA*, 1 had *msrA* and *ermB*, 4 had *msrA*, *ermA* and *ermC*, 1 had *msrA* and *ermC* genes. A total of 15 isolates were negatives for all erythromycin resistance genes tested. Among MRSA isolates 64 were intermediate resistant to erythromycin. Of these isolates 36 were positive for *ermA*, 1 isolate had both *ermA* and *ermC*, and 1 isolate was positive for *msrA*. All remaining 26 isolates were negatives for the genes tested. Among 168 lincomycin resistant isolates 9 had *ermA*, 17 had *ermC*, 1 had both *ermA* and *ermB*, 1 had both *ermB* and *ermC*, 124 had both *ermA* and *ermC*, 4 had *msrA, ermA* and *ermC*, 2 had *ermA*, *ermB* and *ermC*, 1 had *linA* and *msrA*, 1 had *ermC* and *msrA* genes found and 8 isolates were negative by PCR for the genes tested. All lincomycin resistant isolates were tested for clindamycin inactivation by Gots’ test and 3 isolates were found to be positive for inactivation. Of these 3 isolates one carried *linA* gene responsible for lincosamide inactivation and also *msrA* gene, but remaining 2 isolates were negatives for both *linA* and *linB* genes.

Among macrolide resistant isolates the most frequently encountered gene was *ermA* (185 isolates) folloved by *ermC* (157 isolates), *msrA* (10 isolates) and *ermB* (9 isolates).

A total of 358 isolates were resistant to gentamycin and 334 of these isolates were positive for *aac-aph* gene and remaining 24 isolates were negative for this gene.

It was found that 350 of 397 isolates were resistant to tetracyclin. Of these 350 isolates 314 carried *tetM* gene and 36 did not carry this gene. Among *tetM* negative 36 isolates 10 had *tetK* gene and remaining 26 isolates were negative both *tetM* and *tetK*. Distribution of resistance genes among resistant isolates are shown in Table 5. Among macrolide resistance isolates the most common gene combination was *ermA*–*ermC*. Among tetracyclin and gentamycin resistant isolates the most common resistant genes were *tetM* and *aac-aph*, respectively.

**Table 5:** **Distribution of resistance genes**

**Number of Resistant isolates (%)**	**Antibiotics**								
	Erythro*	**Linco**	**Tetra**	**Genta**	**Cipro**	**Line**	**F. Acid**	**Vanco**	**Kana**
	**225 (56.7)**	**168 (42.3)**	**350 (88.2)**	**358 (90.2)**	**366 (92.2)**	**0 (0)**	**32 (8.1)**	**0 (0)**	**363 (91.4)**
Gene (%)	*ermA* (21.3)	*ermA* (5.4)	*tetM* (90)	*aac-aph* (93)	ND**	ND	ND	ND	ND
	*ermB* (0)	*ermB* (0)	*tetK* (2.9)						
	*ermC* (8.9)	*ermC* (10.1)							
	*ermA*-B (0.4)	*ermA-B* (0.6)							
	*ermB-C* (0.4)	*ermB-C* (0.6)							
	*ermA-C* (56.9)	*ermA-C* (73.8)							
	*ermA-B-C* (0.9)	*ermA-B-C* ( 1.2)							
	*msrA* (0.9)	*msrA* (0)							
	*msrA,ermA* (0.9)	*msrA,ermA* (0)							
	*msrA, ermB* (0.4)	*msrA, ermB* (0)							
	*msrA,ermC* (0.4)	*msrA,ermC* (0.6)							
*linA, msrA* (0.6)	*msrA,ermA-C* (1.8)	*msrA,ermA-C* (2.4)							
	Unknown (6.6)	Unknown (4.7)	Unknown (7.1)	Unknown (7)					

*Erythro, Erythromycin; Linco, Lincomycin; Tetra, Tetracycline; Genta, Gentamycin; Cipro, Ciprofloxacin; Line, Linezolid; F. Acid, Fusidic Acid; Vanco, Vancomycin; Kana, Kanamycin.

**ND; Not Determined.

## Discussion

Antibiotic resistance became an important public health problem in Turkey as it is in whole world. Restriction of beta lactam use in MRSA isolates required use of other types of antibiotics for the treatment of infections due to MRSA isolates so survey of susceptibilities of MRSA isolates for antibiotics other than beta lactams became very important.

Our study is the largest study done in Turkey which evaluates both phenotypic and genotypic aspect of antimicrobial resistance among MRSA. A study done in Harran University, Urfa at 2004 indicated that erythromycin, clindamycin, gentamycin and ciprofloxacin resistant among MRSA isolates 63%, 50%, 81% and 25%, respectively [13]. Other study done at Manisa, at 2007 evaluated resistance of MRSA isolated from 1998 to 2002 [14]. It was shown that erythromycin resistance decreased from 59.5% to 51%, clindamycin resistance increased from 28.4% to 41.5%, tetracyclin resistance increased from 57.6% to 88%, and gentamycin resistance from 28.4% to% 87.5, ciprofloxacin resistance from 34.1% to 92.2%. Our study confirmed the tendency for increase in the resistance level of ciprofloxacin, tetracyclin and gentamycin. Sarıbas et al. investigated macrolide resistance genes among MRSA isolates and showed that macrolide resistance level was 29.9% and 86% of the resistant isolates carried *ermA* gene [15]. Sarıbaş et al. found resistance level lower than our study and other studies from Turkey however resistance gene profile was similar with 86% of *ermA* gene but in our study more than 50% of *ermA* positive isolates also carried *ermC* gene. Gül et al. evaluated erythromycin resistance rate among MRSA isolated between 2003–2006 and found resistance rate as 84.9% [16]. Of resistant isolates 37.7% had *ermA* 26.6% had *ermC* and 18.6% had both *ermA* and *ermC* [16]. Aktaş et al. studied 22 erythromycin resistant MRSA isolated in Istanbul and found thet the most frequent genotype was presence of both *ermA* and *ermC* 40.9(%) followed by *ermC* (18.2%) [17]. Ardıç et al. also found among 28 erythromycin resistant MRSA that presence of both *ermA* and *ermC* was the most frequent genotype [18].

In the world among erythromycin resistant MRSA isolates ermA was the most frequent gene in France (57.6%) [10], Colombia (78.5%) [19] and Malesia (52.8%) [20] but in Greece which is neighbour of Turkey *ermC* (96.5%) [21] found to be the most frequent gene.

In our study the most frequent mechanism of macrolide resistance among MRSA isolates found to be presence of methylase. Presence of methylase may confer inducible lincomycin resistance which should be taken in consideration for treatment design. The dominant genes among tetracyclin and aminoglycoside resistant isolates were *tetM* and *aac-aph,* respectively.

The dissemination of resistance was also analysed at regional level. İsolates from Istanbul had lower tetracycline resistance than other regions. MRSA isolates from Van had higher macrolide resistance rates than other regions. Ciprofloxacin resistance rates were very high in all centers and the lowest rate was in Trabzon with 74% and highest rates were in Aydın, Ankara and Izmir with 100%. Tetracyclin resistance was lowest in Istanbul with 52.7% and highest in Ankara with 100%. Fusidic acid resistance rates were relatively low. All isolates from Ankara, Aydın and Manisa were susceptible to fusidic acid, and highest resistance rate was in Afyon with 21.7%. Erythromycin resistance was lowest at Van with 4.7% and highest at Konya with 89.3%. At the same time Konya was the center where the resistance rate differences were the highest between erythromycin and lincomycin. Resistance rates were the lowest in Afyon and Samsun with <50%. Lincomycin resistance rate was 46.2%. Resistance to gentamycin was lowest in Trabzon with 68.5% and highest in Istanbul with 96.3%.

In our study the most common gentamycin resistance gene was *aac-aph* gene (96%). Ardıç et al. studied with 17 gentamycin resistant MRSA isolates from Istanbul at 2006 and found 16 of 17 (94,1%) isolate carried *aac-aph* gene [22]. A study from Iran, neighbour state of Turkey, showed that isolates from Tehran *aac-aph* gene was the most common gene among gentamycin resistant *S. aureus* (83%) [23]. Tetracyclin resistance gene *tetM* was 90% positive among tetracyclin resistant isolates which were only 49% among resistant isolates from Malesia [20].

## Conclusion

Our study is one of the largest epidemiological study done in Turkey. These multi-centre data of resistance level and mechanism of resistance of MRSA isolates will be important for future surveillance studies to determine the evolution of resistance levels and mechanisms at national and regional level. Also our results and follow up studies may constitute a database for empirical treatment of infections due to MRSA. Our multicentre study showed that isolates from 12 centres from Turkey had multiple resistances. Quinolone and gentamycin resistance found to be very high. Fusidic acid resistance was low and erythromycin and lincomycin susceptibility found to be relatively high. This study indicated that resistance to linezolid and vancomycin resistance is not emerged among MRSA isolates from Turkish hospitals.
